# Junk Food Marketing on Instagram: Content Analysis

**DOI:** 10.2196/publichealth.9594

**Published:** 2018-06-05

**Authors:** Amy Jo Vassallo, Bridget Kelly, Lelin Zhang, Zhiyong Wang, Sarah Young, Becky Freeman

**Affiliations:** ^1^ Prevention Research Collaboration, School of Public Health Charles Perkins Centre The University of Sydney Sydney, NSW Australia; ^2^ Early Start Research Institute School of Health and Society University of Wollongong Sydney, NSW Australia; ^3^ School of Information Technologies The University of Sydney Sydney, NSW Australia

**Keywords:** food and beverage, advertisements, social media

## Abstract

**Background:**

Omnipresent marketing of processed foods is a key driver of dietary choices and brand loyalty. Market data indicate a shift in food marketing expenditures to digital media, including social media. These platforms have greater potential to influence young people, given their unique peer-to-peer transmission and youths’ susceptibility to social pressures.

**Objective:**

The aim of this study was to investigate the frequency of images and videos posted by the most popular, energy-dense, nutrient-poor food and beverage brands on Instagram and the marketing strategies used in these images, including any healthy choice claims.

**Methods:**

A content analysis of 15 accounts was conducted, using 12 months of Instagram posts from March 15, 2015, to March 15, 2016. A pre-established hierarchical coding guide was used to identify the primary marketing strategy of each post.

**Results:**

Each brand used 6 to 11 different marketing strategies in their Instagram accounts; however, they often adhered to an overall theme such as athleticism or relatable consumers. There was a high level of branding, although not necessarily product information on all accounts, and there were very few health claims.

**Conclusions:**

Brands are using social media platforms such as Instagram to market their products to a growing number of consumers, using a high frequency of targeted and curated posts that manipulate consumer emotions rather than present information about their products. Policy action is needed that better reflects the current media environment. Public health bodies also need to engage with emerging media platforms and develop compelling social counter-marketing campaigns.

## Introduction

### Background

One of the most significant and preventable causes of poor health and early death is the rapidly rising rates of overweight and obesity. Excess adiposity is a major risk factor for a number of chronic conditions, including cardiovascular disease, type 2 diabetes, osteoarthritis, and some cancers [1,2]. In 2010 alone, it was estimated that, globally, overweight and obesity caused 3.4 million deaths [3].

The alarming increase in obesity prevalence worldwide [3] is predominantly driven by dietary changes, with food now increasingly processed, affordable, and aggressively marketed [2]. The majority of food consumed in developed countries is manufactured and distributed by large for-profit companies [4]. A key driver of company food product sales is highly effective and omnipresent marketing. Current evidence demonstrates that acute exposure to food advertising influences dietary choices [4,5] and increases food intake in children [6]. In adolescents and adults, advertising builds substantial brand awareness, particularly for fast food [7].

Young people’s critical assessment of advertising requires the ability to discriminate between commercial and noncommercial content, recognition of the persuasive intent of advertising, and the ability to apply reasonable skepticism to it [8]. There is evidence that children younger than 8 years are not able to recognize the persuasive intent of advertising, and although these skills develop with time, the age children start to recognize these messages is unknown [8]. The advent of social media, which promotes engagement and friendship between brands and consumers, may diminish the ability to discriminate between commercial and noncommercial content [9].

With the rising popularity of social media, brands are using this platform to reach a diverse range of audiences, using a variety of powerful marketing strategies. Importantly and uniquely, social media allows for personalization of advertisements, which increases consumer receptivity of advertising, as it is tailored to their specific needs and interests [10]. Social media users can also communicate with their friends and followers about a brand or product, for example, by tagging a friend, reposting competitions, or tagging brands in their own personal posts. This extends the reach of individual advertisements and creates the sentiment that certain brands and products are endorsed by peers [10] or high-profile social media users, sportspeople, and celebrities [11].

Advertising has a strong influence on food preferences, choices, and consumption, particularly in children [6,8,12]. Adolescents are considered the primary targets for digital marketing [10] because of their own disposable income, spending power, ease of technology use, and role in setting and following trends [13]. Advertisers and food companies often assert that the purpose of their advertising is to provide information about their brands and products, including their healthier options, to allow consumers to make an informed choice about purchase and consumption [14]. It is, therefore, vital to gain a better understanding of how energy-dense, nutrient-poor (EDNP) food and beverages are being advertised on popular social media platforms and if the claims about the supposed purpose of advertising are justified.

Instagram is a popular mobile photo-sharing and, more recently, video-sharing social media platform. It was launched in October 2010, purchased by Facebook in 2012 [15], and in April 2017, there were more than 700 million monthly active users worldwide [16]. In 2015, Instagram was the most used social media platform among teenagers in the United States [17]. In 2016, it was the second most popular social media platform (after Facebook) among those aged older than 18 years [18] and more popular than Facebook in certain ethnic groups [19]. Many businesses have established their own accounts, as a growing number of people are using social media to “follow or find out about particular brands or businesses” [20]. In March 2017, Instagram reported that 8 million companies used the social media site—a rapid increase from the 1.5 million companies active on the platform in September 2016 [21].

Previous research has investigated how “junk” food and nonalcoholic beverages are promoted on Facebook [22] or the effects of user-generated images [23], and highlighted that a larger study is required to better understand the promotion elements used by brands on Instagram [24]. The engagement rate between advertiser and user is higher for Instagram than Facebook [25], indicating that, although fewer people have an Instagram account, there is potentially more total activity between brands and consumers. As Instagram was designed as a mobile app, it is frequently and repeatedly accessed. For example, globally, at least 60% of users log in daily [26], and in countries such as Australia, users check their accounts multiple times per day and an average of 26 times per week [20], which again demonstrates the potential for high reach of branded messages and normalize behavior [27].

### Objectives

Due to the great potential of social media to influence the food and beverage choice of young people, it is essential to understand how brands are advertising their products online. Therefore, the primary aim of this study was to investigate the frequency of images and videos posted by the most popular ENDP food and beverages brands on Instagram and the marketing strategies used in these images. Secondarily, the study also aimed to evaluate if posts contained any product information, health claims, or healthier choice claims.

## Methods

### Selection of Instagram Accounts

A total of 15 of the most popular ENDP food and beverages accounts on Instagram were included. These were Ben and Jerry’s, Burger King, Coca-Cola, Dominos, Gatorade, KFC, McDonald’s, Monster Energy, Nutella, Oreo, Pepsi, Red Bull, Starbucks, Subway, and Taco Bell. Popular ENDP brands (excluding grocery stores) were first identified based on 2014 global sales rankings and checked for the presence of an Instagram account. If the account had at least 100,000 followers (in November 2015), it was included in the study (16 brands in total). For brands that maintained both global and nation-specific Instagram accounts, the global account was included. For each included brand, 12 months of Instagram posts from March 15, 2015, to March 15, 2016, were extracted, including the image, caption, number of likes and number of comments for each image, and for videos, the number of views. One brand (Wendy’s) was then excluded from the study, as its first Instagram post was on July 10, 2015, which was partway through the study period.

### Establishing the Coding Guide

A coding guide was used to identify the primary marketing strategy of each post. The first iteration of the guide was based on the categories developed in previously published works, including branded characters, branding elements, celebrities, children’s characters, competitions, corporate social responsibility or philanthropy, engagement, links, special price promotions, sponsorships or partnerships, sportspeople, videos, and vouchers [22,28]. This guide was then used to code a subsample of 10 images from each Instagram account included in this study. If an image could not be suitably coded into any of the existing categories, a new category was added in an iterative process. This was done to ensure the coding frame was suitable for Instagram content. Two new marketing categories emerged: product imagery and image with no clear marketing strategy (which included images unrelated to the brand or product, for example, the view from an employee’s window).

The coding guide also contained instructions to code each post regarding whether it contained content that was informational (yes or no), original (yes or no), or portrayed the products as healthy choices or contained health claims (yes or no). Table 1 contains a summarized version of coding guide categories and definitions.

For each post, an indicator of marketing strategy was recorded. Where more than one marketing strategy featured in a single post, strategies were coded according to the following hierarchy: Corporate Social Responsibility or Philanthropy>Celebrities or Sportspeople or Children’s characters or Branded characters>Special price promotions or vouchers or competitions>Engagement>Sponsorships or Partnerships>Videos>Links>Branding>Product imagery. For example, if a post contained a video of a celebrity holding a branded product, it was coded as Celebrity. The primary rationale in establishing this hierarchy was to ensure consistency between coders, rather than to imply one marketing strategy is more important or meaningful than another. However, the strategies that were anticipated to be the most common (eg, branding) were placed at the bottom of the hierarchy in an attempt to capture the breadth of marketing strategies used on Instagram. The informational content, original and health claim categories were not hierarchical, and each post was also assessed against these criteria.

### Coding the Images

Two authors (AJV and SY) were responsible for coding each image. First, one complete account (Ben and Jerry’s) was coded by both coders independently. All codes assigned for all images were checked for agreement, and any discrepancies were discussed and agreed upon, incorporating the opinion of a third coder (BF) if required. All other accounts were then coded by one coder each (AJV or SY). If coders were unsure how to categorize a post, it was discussed and coded together, again incorporating a third coder (BF) if required. Descriptive comments about key features of each account were also recorded narratively during the coding process.

**Table 1:** Definitions of marketing strategies and coding criteria.

Category		Definition
**Assess each post for**		
	Informational content	Recognizable information about what the product looks/tastes like, ingredients, or place of purchase
	Original (brand-generated) content	Image is generated by the brand, or third party sponsored by brand, as opposed to consumer
	Health claims	Specific reference that the product shown is a healthy choice or may improve physical health
**Code marketing strategy**		
	Corporate social responsibility or philanthropy	Statement of ethical or sustainable standpoint or initiative or charitable work undertaken by brand
	Celebrities	People with an entertainment or media profile, excluding athletes
	Sportspeople	Any person showing their athletic ability and/or sporting achievements, including extreme and motorsports
	Children’s characters	Third-party cartoons or characters, including characters from films, books, TV, and the internet
	Branded characters	Any characters developed by the brand
	Special price promotions	Limited time offers, discount menus, 2 for 1 deals, or other reduced price advertisements
	Vouchers	“Offers” exclusively available to those who like the account, including print off and/or electronic codes
	Competitions	Any contest involving participant entry, including minimal requirements, for example, liking a post
	Engagement	Posts that prompt interaction/conversation
	Sponsorships or partnerships	Any events the brand supports or brands/service partners, excluding charitable organizations (coded as corporate social responsibility, see previously)
	Videos or Graphics Interchange Format (GIFs) or boomerang	Moving images
	Links	Link to an external page or additional content
	Branding elements	Logos, colors, fonts, trademarks, or slogans
	Product imagery (unbranded)	Pictures of the products sold or their ingredients, with no labels or branding elements
	Image with no clear marketing strategy	Image not associated with any other marketing category

When determining which codes to assign, post elements were examined systematically. First, the image was viewed, and then, if the primary marketing strategy could not be determined based on image alone, the original image caption was read (the first comment found under an Instagram post and made by the account owner). Any further comments by followers or the account owner were not incorporated.

### Analysis

Interrater reliability was established by both coders independently coding the same random sample of 10 posts from each account (excluding Ben and Jerry’s, n=140). A percentage agreement score was then calculated between the 2 coders. Percentage agreement of 80% or above was considered acceptable.

Descriptive statistics were generated in Excel (Microsoft) for each individual account for the average number of posts, average number of post likes and comments, and average number of views of videos (views are not published for image-based posts). Proxy interaction between account and consumer was determined by calculating the percentages of followers actively interacting with the account via liking or commenting on posts, or passively interacting by viewing videos. Instagram counts a view when a video is watched for at least 3 seconds. Viewing data were only available for videos posted after November 20, 2015, as this was when Instagram began releasing this information. All videos containing viewing data were included in this aspect of the analysis, not just those that were coded as video for their primary marketing strategy. The most common marketing strategies and the range of strategies used by each account were also determined.

## Results

### Brand Characteristics

Of the 15 Instagram accounts included in the study, there was one ice cream brand (Ben and Jerry’s), one sweet biscuit (Oreo), and one sweet spread (Nutella), which were combined into a sugary grocery store foods category. There were also 2 soft drink brands (Coca-Cola and Pepsi), 3 energy drinks (Gatorade, Monster Energy, and Red Bull), and 7 fast food chains (Burger King, Dominos, KFC, McDonald’s, Starbucks, Subway, and Taco Bell). The characteristics of each Instagram account, such as page likes and number of posts during the study period, are summarized in Table 2.

At the time of the study, Starbucks was the most popular account with 6.6 million followers, and Subway was the least popular in our sample with 109K followers. There was a large variation in the number of posts by each account, with some accounts (Dominos, Monster Energy, and Red Bull) posting multiple times a day, and other accounts (Oreo, Subway, and Pepsi) only posting every 3 to 4 days.

**Table 2:** Characteristics of top food and beverage Instagram accounts.

Rank	Account name and Instagram handle	Followers, n (Nov 2015)	Posts during study period, n	Average likes per post	Followers who liked post, %	Average comments per post	Followers who commented, %	Videos with views data, n	Average views per video	Followers who viewed video, %
1	Starbucks Coffee	6.6M	312	204,833	3.10	1318	0.02	10	518,903	7.86
2	Red Bull	3.8M	419	74,261	1.95	803	0.02	38	380,592	10.02
3	Monster Energy	2.6M	685	36,888	1.42	178	0.01	61	101,293	3.90
4	OREO	1.1M	94	31,510	2.86	628	0.06	20	110,828	10.08
5	McDonald’s	1M	128	21,978	2.20	806	0.08	11	104,730	10.47
6	Coca-Cola	920K	117	19,333	2.10	317	0.03	24	53,150	5.78
7	Gatorade	678K	226	12,489	1.82	82	0.01	8	34,306	5.06
8	Taco Bell	671K	183	18,036	2.69	810	0.12	7	71,454	10.65
9	KFC	632K	160	7,697	1.22	235	0.04	8	30,884	4.89
10	Ben and Jerry’s	588K	295	17,470	2.97	609	0.10	6	50,180	8.53
11	Burger King	530K	186	9,666	1.82	411	0.08	2	30,628	5.78
12	Domino’s Pizza	431K	407	5,722	1.33	159	0.04	2	32,078	7.44
13	Pepsi	320K	136	4,545	1.42	610	0.19	9	15,523	4.85
14	Nutella	301K	226	14,260	4.74	373	0.12	16	66,150	21.98
15	Subway	109K	98	2,025	1.86	113	0.10	3	12,313	11.30
Average of all brands		1,352,000	245	32,048	2.37	497	0.04	15	107,534	7.95

**Table 3:** Marketing strategies used by brands on Instagram.

Instagram account		Top 3 most used marketing strategies (posts)			Marketing strategies (N=15), n	Informational posts, n (%)	Original posts, n (%)	Health claims, n
		1	2	3				
**Sugary grocery store foods**								
	Ben and Jerry’s (n=295)	Branding (123)	Links (66)	Corporate/social responsibility (38)	10	251 (85.1)	255 (86.4)	1
	Nutella (n=226)	Branding (152)	Product image (65)	Competition and engagement (3)	6	219 (96.9)	218 (96.5)	0
	Oreo (n=94)	Product image (48)	Branding (23)	Videos (11)	8	81 (86)	91 (97)	0
**Soft drinks**								
	Coca-Cola (n=117)	Branding (51)	Videos (34)	Children’s characters (17)	8	70 (59.8)	92 (78.6)	1
	Pepsi (n=136)	Branding (88)	Competition (19)	Videos (19)	8	84 (61.8)	124 (91.2)	0
**Energy drinks**								
	Gatorade (n=226)	Sportspeople (124)	Branding (77)	Image with no clear strategy (12)	7	96 (42.5)	226 (100)	9
	Monster Energy (n=685)	Sportspeople (552)	Branded character (59)	Branding (28)	9	20 (2.9)	676 (98.7)	3
	Red Bull (n=419)	Sportsperson (310)	Branding (31)	Videos (25)	11	15 (3.6)	416 (99.3)	0
**Fast food chains**								
	Burger King (n=186)	Branding (99)	Product image (59)	Partnerships (17)	8	169 (90.9)	165 (88.7)	0
	Dominos (n=407)	Product image (232)	Branding (101)	Image with no clear strategy (33)	11	302 (74.2)	407 (100)	4
	KFC (n=160)	Branding (45)	Branded characters (26)	Videos (22)	10	91 (56.8)	160 (100)	0
	McDonald’s (n=128)	Branding (56)	Product image (35)	Videos (22)	7	108 (84.4)	119 (93.0)	0
	Starbucks (n=312)	Branding (175)	Product image (67)	Links (26)	10	242 (77.6)	228 (73.1)	2
	Subway (n=98)	Branding (50)	Product image (29)	Special price promotion (6)	6	70 (71)	97 (99)	2
	Taco Bell (n=183)	Branding (88)	Product image (45)	Special price promotion (11)	11	140 (76.5)	182 (99.5)	1

Only a small percentage of followers actively interacted with the branded accounts via liking (1.22%-4.74%) or commenting (<1%) on their posts. Since November 2015, viewing data is publically available for video posts only, and this gives some indication of the level of passive interaction between consumers and branded accounts. Within each Instagram account, a greater percentage of followers passively interacted with account videos (up to 21.98%), compared with actively liking or commenting on posts. However, some accounts only had a very small number of videos with views data within the study period, eg, Burger King and Dominos with 2 video posts each. From the descriptive data in Table 2, there also appears to be little relationship between frequency of posts and level of passive or active interaction between account and account followers.

### Marketing Strategies

Interrater reliability for the coding of Instagram posts was acceptable, with percentage agreement scores for accounts coded by individual coders ranging between 80% and 100% for all categories. A summary of marketing strategies and other characteristics of images posted are summarized in Table 3, and the full results of the marketing categorization for all images are in Multimedia Appendix 1.

Each brand used multiple primary marketing strategies in their Instagram accounts, ranging from 6 to 11 different strategies each. Some brands, particularly energy drinks (Gatorade, Monster Energy, and Red Bull), predominately favored certain strategies, with up to 81% of their posts featuring sportspeople. There was also a high level of branding contained in posts, with branding featuring as 1 of the top 3 marketing strategies for each account in this study, although this category was the second last in the coding hierarchy. Use of children’s characters was 1 of the top 3 marketing strategies of just one brand, Coca-Cola.

The level of informational content provided on Instagram was highly variable by brand, ranging from 2.9% to 96.9% of images for each account. Energy drink brands had the lowest number of posts containing product or brand information, and sugary grocery food, particularly Nutella, products had the highest.

The majority of Instagram posts featured original images taken by the brand or a contracted photographer (73.1%-100% of images of each account). There were few identified “regrammed” images, where a brand reposts an image taken by a brand consumer and credits (or “tags”) the image back to them.

There were very few health claim–related images posted on Instagram. Exceptions to this were Subway and Dominos occasionally highlighting the salad components of their product; Starbucks focusing on the fruit content of their smoothies; and Gatorade emphasizing the thirst quenching, electrolyte, and rehydration qualities of their energy drinks. “Healthy alternative” menu items featured occasionally but not prominently in any of the Instagram accounts studied.

### Case Study Examples

Three case studies are presented for the accounts with the most followers (Starbucks), the most posts (Monster Energy), and the greatest amount of passive and active interaction (Nutella).

**Figure 1:**
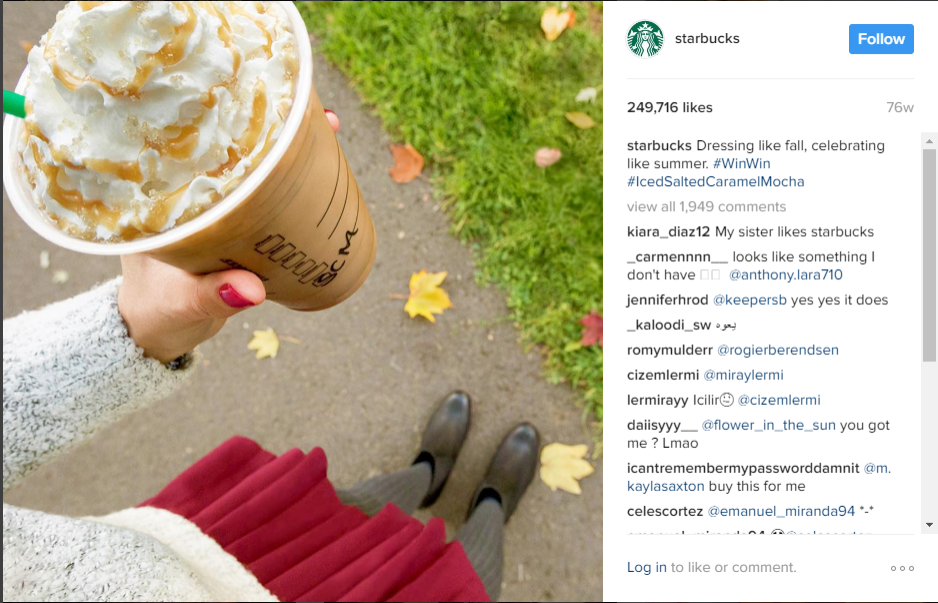
Starbucks example image.

Starbucks contained the highest percentage of regrammed images and images of everyday people consuming their products. In comparison with other accounts, Starbucks was relatively informational about the brand and the products they sell. Figure 1 is a typical Starbucks post and contains a relatable consumer holding an energy-dense product with the highly recognizable Starbucks cup.

**Figure 2:**
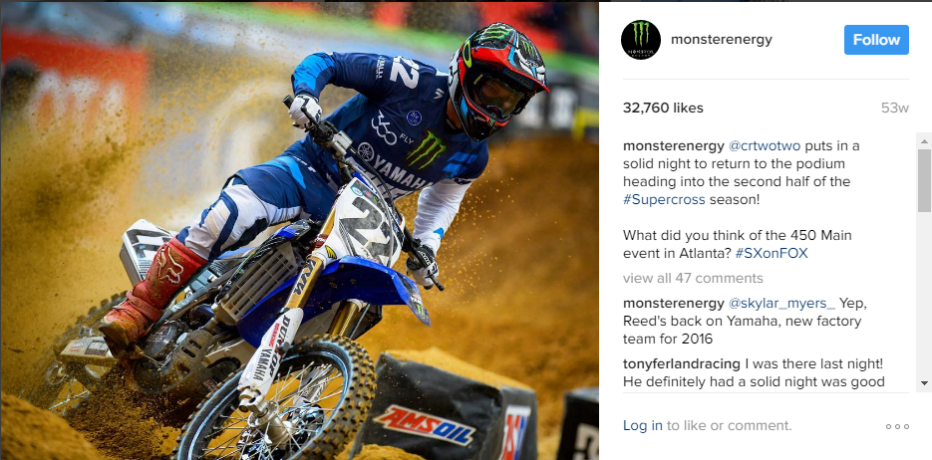
Monster Energy example image.

Monster Energy posted multiple times a day during the study period. Like other energy drinks, this account predominately featured posts of sportspeople and athletic endeavors (Figure 2). This figure shows a typical post by Monster Energy and emphasizes high energy, fiery, and adventurous associations with the brand, which are appealing to the target audience of male millennials [29], but does not feature the beverage itself. Monster Energy had the lowest amount of informational posts (2.9%). However, the Monster branding “M” or colors frequently featured.

**Figure 3:**
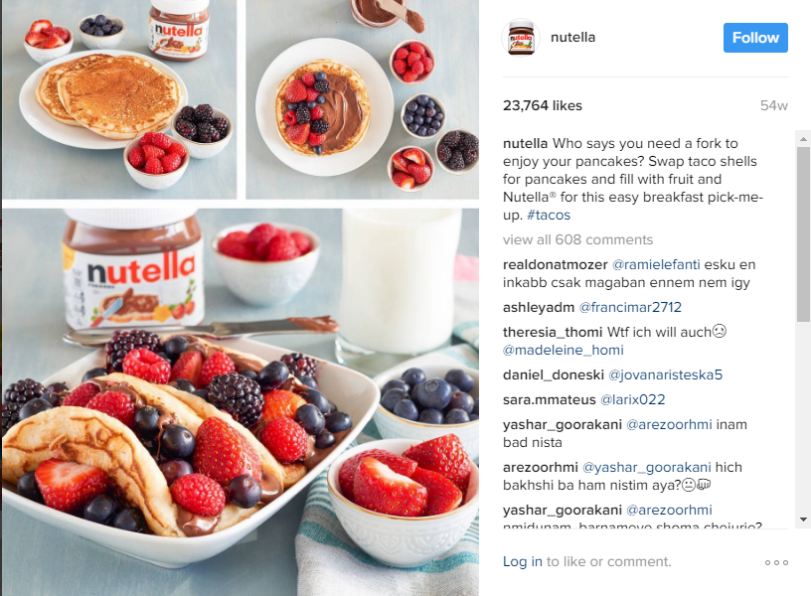
Nutella example image.

Nutella was the brand with the greatest amount of active interaction through followers liking or commenting on their images or viewing their videos. Nutella (Figure 3) also had the greatest percentage of posts that contained consumer information, such as what the product looks like or how to use it (96.9% of posts). Almost all their posts featured different ways to prepare and eat Nutella, as seen in this example image, demonstrating its perceived versatility and the many ways the product can be incorporated into a consumer’s everyday diet. The terms breakfast and start the day were common in Nutella posts, as seen in this caption, as well as incorporation of fruit.

## Discussion

### Principal Findings

This study demonstrates the widespread use of Instagram to market ENDP food and beverages to consumers. The percentage of posts containing any informational content ranged from 2.9% to 96.9%, depending on the brand. Our definition was generous and included any information about the product, what it looks or tastes like, its ingredients, or how it could be purchased. We did not assess quality of the information nor did we find any evidence that brands were sharing important nutritional information about caloric energy, sugar, salt, and fat content, or serving size. In addition, this information was accompanied by an image or video that also included persuasive marketing strategies. Energy drinks, in particular, had very low numbers of informational posts and instead focused on the symbolic uses of their products via extreme sports, rather than including any information on what their product actually is, looks, or tastes like. These results support the conclusion that advertising places less emphasis on communicating specific product information and more on communicating the social and symbolic uses of products [14] and building relationships with consumers [24].

Similar to other marketing platforms, branded characters, symbols, and colors were used throughout the Instagram posts for all brands included in this study. Previous research has found that familiar and/or character branding has a powerful influence on children’s preferences, choices, and intake of foods, especially unhealthy foods [30]. Celebrity endorsement is a common and longstanding strategy used in many advertising platforms [11]. This study found that athletes and celebrities were commonly incorporated into Instagram images, particularly by energy drink brands. Celebrities also have their own Instagram accounts, which are used to promote particular brands through personal testimonies [31], further expanding the reach and persuasiveness of brand advertising, although these pages were not analyzed in this study.

The relatively small percentage of followers who liked or commented on posts demonstrated potentially low levels of active interaction initiated by consumers with brands on Instagram. This aligns with previous research demonstrating that many on social media are passive users who view content but do not actively engage [28]. Yet, despite this, the frequency of posting on Instagram as well as the ability to sponsor, promote, or search for posts and hashtags [32] suggests that consumers may be exposed to a large amount of EDNP food images, especially if they follow multiple big brands and login multiple times a day [33].

This study found a low prominence of healthy products among the most popular brands and few health-related claims on Instagram. Although brands are not directly advertising their products as healthy, they do imply that their products are part of a balanced diet. For example, Nutella commonly uses hashtags like breakfast, or statements like Nutella for breakfast never goes out style. The brands under study also did not promote their healthy options on Instagram. Companies, in particular fast food restaurants, have previously stated that their healthy options are priority products [34]; however, the results of this study do not support this claim. Use of social media also allows companies to bypass promises regarding advertising to children and young people. For example, a spokesperson for McDonald’s has previously commented that the brand takes its responsibility as an advertiser seriously and that they only promote healthier options when communicating to children [35]. Although this may be the case for television advertising during children’s programs, these promises are not being adhered to on Instagram, a finding similar to previous research relating to alcohol marketing to youth [36]. Although Instagram’s terms of use require users to be aged older than 13 years, and therefore brands are operating within the terms of these responsibility statements, statistics from the United Kingdom show that in 2016, approximately 50% of children aged 8 to 15 years use this social media platform [37].

The unique, individual, and highly curated nature of each Instagram account included in this study demonstrates that different audiences are being targeted through these different accounts. Market research has shown that consumers, particularly those of the digital age, are more receptive to advertising that is tailored to their needs and interests and inserted into their personalized media experiences [10]. This exemplifies the extraordinary potential of Instagram and other visually based social media platforms to effectively communicate with potential consumers and encourage regular purchase and consumption of unhealthy foods and beverages. The everyday nature of these posts and constant exposure to these images have the potential to condition consumers to disregard the sometimes or special occasion recommended use of these products and incorporate them as a normal or everyday part of their diet. There are also examples of chains developing new EDNP products based primarily on their photogenicity and ability to trend on Instagram. One example is the Starbucks Unicorn Frappuccino, with a Starbucks spokesperson stating that “the look of the beverage was an important part of its creation, our inspiration came from the fun, spirited and colorful unicorn theme food and drinks…trending on social media” [38].

### Study Limitations

This study has some limitations. First, it was not possible to determine the number of views for each post, which would be valuable information to determine the reach of ENDP food advertising on Instagram. This information is only available to the account owners. In addition, percentage of followers was used in this study as a proxy measure for interaction between branded accounts and the public. However, because of Instagram’s undisclosed algorithm for determining which images appear in what order on a user’s newsfeed, individuals may not see every post from a branded account even if they follow them. Overall, these presented results are likely to be an underestimate of true exposure to these images, as nonfollowers are also able to view these posts through sharing, searching, hashtags, or the more recent Instagram explore feature. Only one primary marketing strategy was assigned to each Instagram post, as coding all applicable strategies to each image would have overcomplicated and reduced the meaningfulness of the results. Therefore, this study presents an underestimate of the use of some marketing strategies, particularly those toward the bottom of this hierarchy, and prevalence of the use of each strategy cannot be accurately reported. Moreover, this study only analyzed images posted on each brand’s account; it did not incorporate paid advertisement or promoted posts, which appear on users’ newsfeed whether or not they follow that brand. Finally, any assessment of the followers of each Instagram account was not incorporated into this study and is instead a topic for future research in this field.

### Conclusions

Instagram is a social media platform where the promotion of EDNP foods and beverages is flourishing. This study has demonstrated a high frequency of advertising by top food and beverage brands on Instagram through targeted and curated posts using a range of marketing strategies. Brands are using social media platforms such as Instagram to advertise to a growing number of consumers, with little to no restrictions. Public health bodies need to act to develop compelling social counter-marketing campaigns as a way of combating this advertising [39]. This study also contributes to the growing evidence of the urgent need to establish policy action regarding ENDP food and beverage advertising [40] that is reflective of the current media environment.

## Appendix

Multimedia Appendix 1 All coded data for marketing strategies used by brands on Instagram.

## Abbreviations

EDNP: energy-dense, nutrient-poor
