# Supplementary material for: Junk Food Marketing on Instagram: Content Analysis
Source: JMIR Public Health Surveill. 2018 Jun 5;4(2):e54. doi: 10.2196/publichealth.9594 (PMC6008515; doi:10.2196/publichealth.9594)
Supplement: Multimedia Appendix 1 [file publichealth_v4i2e54_app1.pdf]

Multimedia Appendix 1: All coded data for marketing strategies used by brands on Instagram

Account name	Primary marketing strategy (n)															Other post content		
	Competitions	Special price promo	Vouchers	Celebrities	Children's characters	Sports people	Branded characters	Branding elements	Product imagery	Image	Video	Engagement	Links	Sponsorship	CSR / philanthropy	Informational posts (%)	Original posts (%)	Healthy posts (n)
**Grocery store foods**																		
Ben and Jerrys (295 posts)	13	0	0	16	0	0	0	123	15	1	16	1	66	6	38	85	86	1
Nutella (226 posts)	3	2	0	0	0	0	0	152	65	0	1	3	0	0	0	97	96	0
Oreo (94 posts)	1	1	0	0	0	0	0	23	48	1	11	1	8	0	0	86	97	0
**Soft drinks**																		
Coca-Cola (117 posts)	1	0	0	0	17	0	0	51	6	0	34	4	2	0	2	60	79	1
Pepsi (136 posts)	19	0	0	1	0	0	0	88	0	1	19	0	1	6	1	62	91	0
**Energy drinks**																		

Gatorade (226 posts)	0	0	0	0	0	124	0	77	2	12	8	0	2	0	1	43	100	9
Monster Energy (685 posts)	0	0	0	2	0	552	59	28	0	13	11	1	18	1	0	3	99	3
Red Bull (419 posts)	7	0	0	10	4	310	0	31	0	3	25	4	3	12	10	4	99	0
**Fast food chains**																		
Burger King (186 posts)	1	4	0	1	0	0	2	99	59	0	3	0	0	17	0	91	89	0
Dominos (407 posts)	7	0	1	0	0	3	0	101	232	33	14	4	7	2	3	74	100	4
KFC (160 posts)	1	0	0	0	0	4	26	45	16	21	22	0	5	18	2	57	100	0
McDonalds (128 posts)	0	0	0	0	0	1	0	56	35	11	22	2	0	1	0	84	93	0
Starbucks (312 posts)	9	16	0	0	0	0	0	175	67	3	4	4	26	1	7	78	73	2
Subway	0	6	0	1	0	1	0	50	29	5	3	2	0	1	0	71	99	2

(98 posts)																		
Taco Bell<br><br>(183 posts)	4	11	0	1	0	0	0	88	45	1	4	6	9	7	7	77	99	1
